# Intracranial Masson's Tumor: A Case Report

**DOI:** 10.7759/cureus.71860

**Published:** 2024-10-19

**Authors:** Merih C Yilmaz, Önder Taşkın, Salih B Yilmaz, Zehra Akman

**Affiliations:** 1 Neurosurgery, VM Medical Park Samsun Hospital, Samsun, TUR; 2 Neurological Surgery, Amasya Kolmed Hospital, Amasya, TUR; 3 Pathology, Republic of Türkiye Ministry of Health SBU (Sağlık Bilimleri Üniversitesi) Van Education and Research Hospital, Van, TUR

**Keywords:** cranioplasty, hyperplasia, intracranial, intravascular papillary endothelial hyperplasia (ipeh), masson’s tumor, vascular lesion

## Abstract

This case report presents a unique instance of Masson's tumor, highlighting its atypical presentation and diagnostic challenges. A 19-year-old male patient underwent cranioplasty surgery after presenting with swelling on the frontal scalp. No history of trauma was reported. We compared the preoperative and postoperative results and diagnosed an intracranial Masson's tumor pathologically, which was not detected radiologically. Ensuring complete excision of Masson's tumor stands as the foremost imperative for averting relapse, underscoring the significance of meticulous attention to the aesthetic outcome in cases undergoing concurrent cranioplasty surgery. By elucidating the correlation between the tumor and the patient's symptoms, this report contributes valuable insights into the clinical manifestations and management of this rare intracranial pathology. This addition to the scientific literature underscores the importance of comprehensive evaluation and tailored therapeutic approaches for such cases, ultimately enriching our understanding of Masson's tumor and its clinical implications.

## Introduction

Masson's tumor is the commonly used term for intracranial intravascular papillary endothelial hyperplasia (IPEH). It was initially documented by histopathologist Pierre Masson in 1923. IPEH is an exceptionally uncommon condition. Masson’s tumor is a rare, benign vascular lesion that typically develops within a pre-existing thrombus or vascular malformation. This pathology is more frequently found in the extremities of the body, affecting subcutaneous and cutaneous tissues [1].

Masson's tumor is a vascular lesion distinguished by the development of papillary structures lined with endothelial cells and containing collagenous cores [2]. Extracranial IPEH usually presents as a slowly growing painful nodule. Intracranial IPEH has far from specific clinical symptoms and may present with intracranial mass symptoms and neurologic deficits. Masson tumor is an endothelial, non-neoplastic proliferation. Recurrence may occur after subtotal resection [3,4].

While commonly found in the extremities, the intracranial occurrence of Masson's tumor is extremely rare. This report provides an overview of its unusual intracranial manifestation and the clinical challenges associated with diagnosis and treatment.

## Case presentation

**Figure 1:**
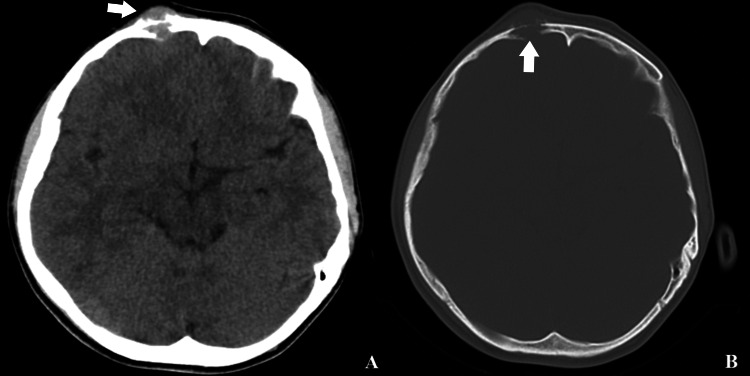
Preoperative computed tomography of the brain showing parenchymal (A) and bone window (B) view of the lesion that erodes the bone and gives an expansile appearance to the skin (White arrow)

A 19-year-old male patient presented with a complaint of a mass in his skull that had been present for three months and was progressively enlarging. There was no definitive history of trauma. A neurological examination revealed no abnormalities. In the computed tomography images of the patient, an 18x18 mm lesion was identified that had eroded the inner and outer tabula of the right frontal bone and caused epidural compression (Figure 1). The patient was thoroughly informed about the surgical procedure and consent was taken prior to the operation, demonstrating a clear understanding and agreement regarding the details and implications of the surgery.

**Figure 2:**
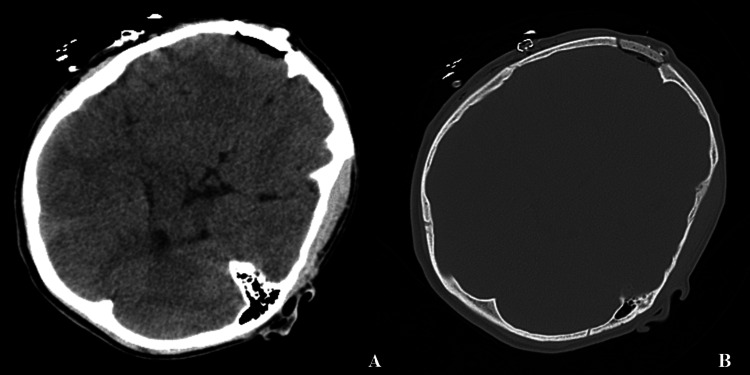
Postoperative computed tomography of the brain after total removal of the lesion and cranioplasty

The patient, prepared for surgery, was positioned supine, and a curved incision was made, ensuring it did not extend beyond the hairline. Upon making the skin incision, it was noted that the lesion had enlarged the bone and infiltrated the scalp. A craniectomy was performed around the healthy bone adjacent to the lesion, during which the purple lesion within the bone was excised. Cranioplasty was then carried out using methyl methacrylate (Figure 2).

Pathological examination was evaluated as Masson's tumor. The patient did not develop postoperative complications and was discharged on the seventh day after skin sutures were removed.

**Figure 3:**
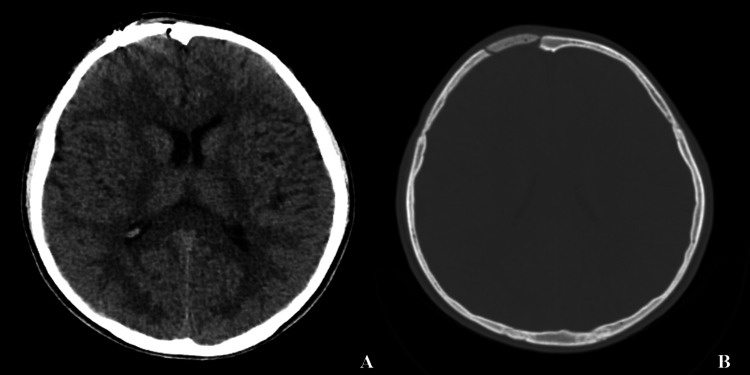
Computerized tomography of the brain at the three-month follow-up showing no recurrence

The neurological examination of the patient at the third-month month outpatient clinic follow-up was evaluated as normal, and no recurrence was detected in the CT taken on the occasion (Figure 3). The patient is undergoing ongoing clinical and radiological follow-up. 

Histopathology

**Figure 4:**
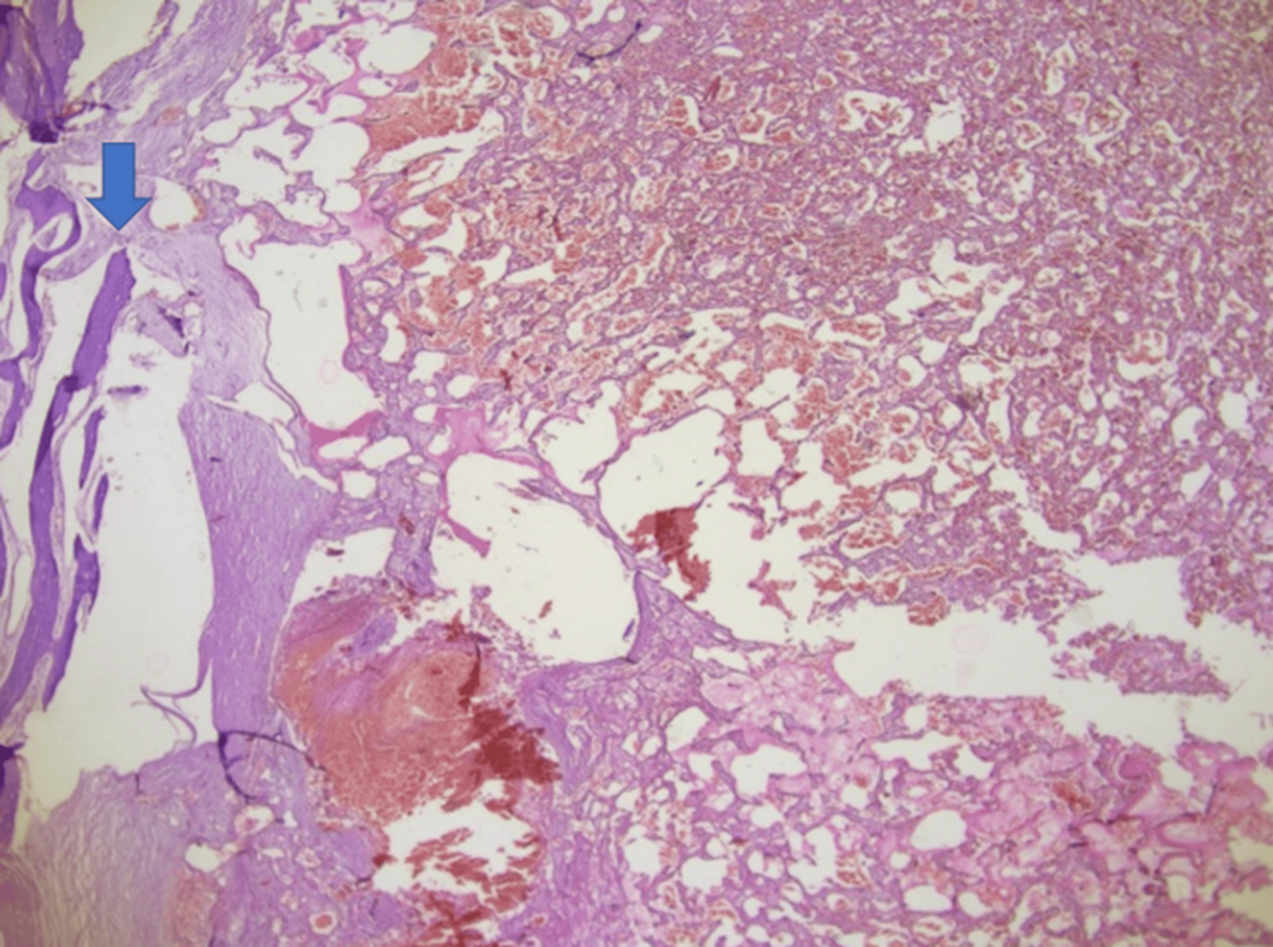
Multiple areas of the tumor show features of intravascular papillary endothelial hyperplasia. Anastomizing vascular lesion destroying bone tissue (blue arrow); hematoxylin & eosin, x200

**Figure 5:**
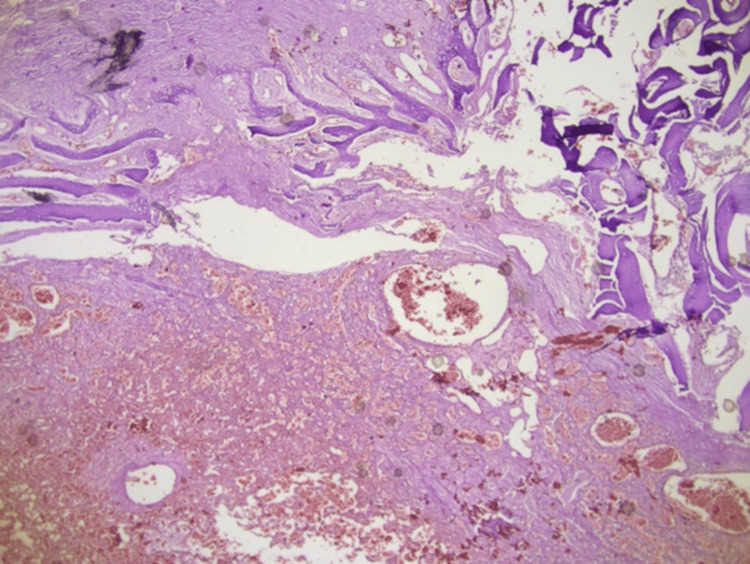
Vascular structures anastomosing within the bone tissue are observed; hematoxylin & eosin, x40

Microscopic examination revealed a vascular lesion with a thrombus-like formation in a focus lined with endothelial cells with diffuse hemorrhage, destroying bone tissue, forming intravascular papillary-like structures in some areas, and hyalinized core structures (Figures 4, 5).

**Figure 6:**
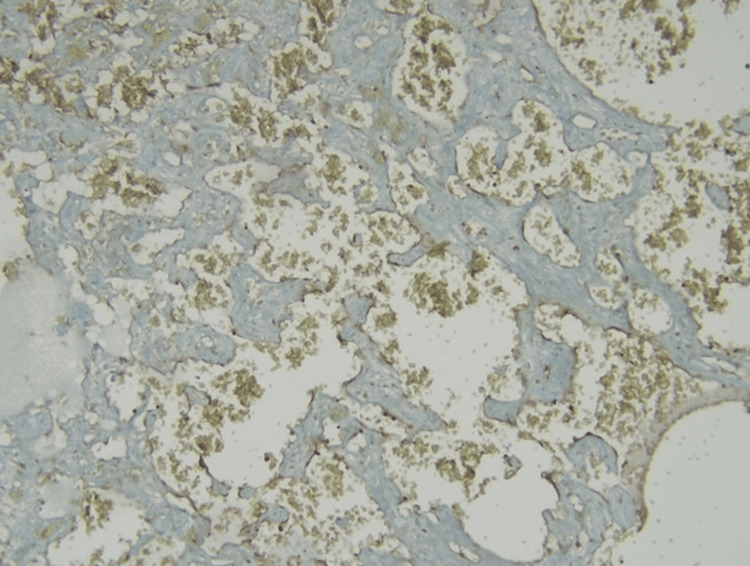
Low Ki-67 proliferation index in papillary structures lined with endothelium with hyalinized cores; Ki-67, x400

**Figure 7:**
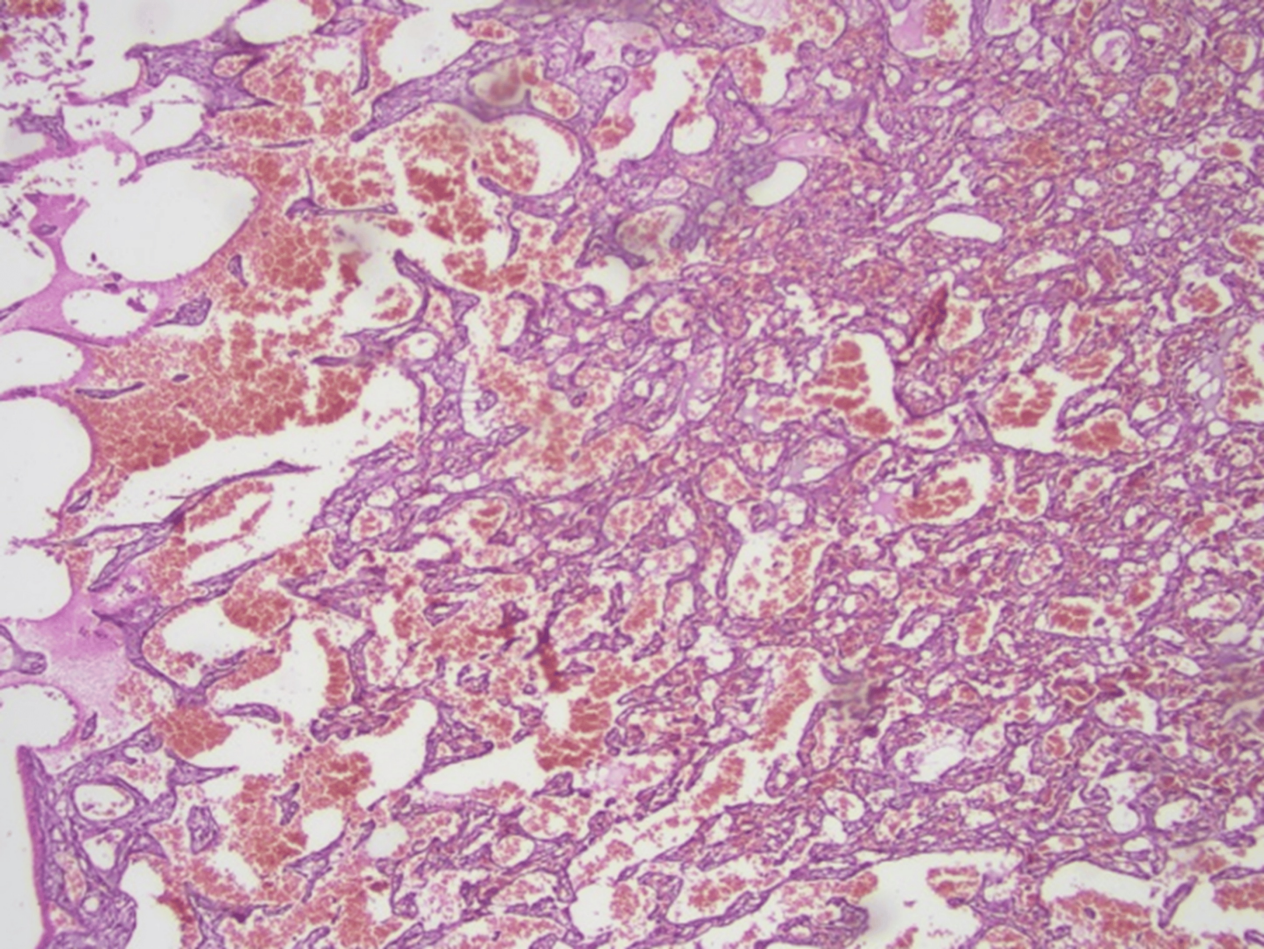
Vascular structures lined with single-layer endothelium, free of atypia and mitosis are observed; hematoxylin & eosin, x100

Significant atypia and necrosis were not observed. In the immunohistochemical examination, staining with cluster of differentiation (CD) 31 and CD34 was observed in the vascular endothelium. No increase in Ki67 (Antigen Kiel 67) proliferation index was observed (Figures 6, 7).

## Discussion

Intracranial Masson's tumor manifests with a range of symptoms primarily determined by the lesion's location, such as indications of local compression and elevated intracranial pressure [5]. Masson's tumor can arise within an existing vascular malformation or thrombus, including locations such as the cavernous sinus, meninges, torcular sinus, superior orbital fissure, internal acoustic canal, cerebellopontine angle, and aneurysms [6-9]. It is typically seen as an atypical arrangement of arterial and venous thrombi or as an incidental microscopic discovery within a hemangioma, instead of as a neoplastic alteration [5,10].

Although it is difficult to distinguish Masson's tumor from angiosarcoma histopathologically, angiosarcoma develops from extravascular structures while Masson's tumor develops from intravascular structures. In a multicentric cohort study by Yang et al., Masson's tumor showed pure-, mixed-, and low-rate extravascular types [11]. In the same study, Masson's tumor stained with immunohistochemical markers such as CD31, CD34, actin, and the relevant gene specific to erythroblast transformation led to an awareness of the differential diagnosis.

In the report by Miyamoto et al. that reviewed over 30 cases, the ages of the patients ranged from eight to 82 years [12]. In their study, Hashimoto et al. studied a total of 91 cases and found that the incidence in women is higher than in men [13]. The surgical aim for Masson's tumor is total resection to prevent recurrence. Recurrence is inevitable in subtotal resections and this has been seen in the literature [13]. Long-term clinical and radiological follow-up is recommended after surgery; Yaduz et al. [14], Sekmen [15], and Moon et al. [16] had follow-ups for 18 months, five years, and nine months, respectively. In the study by Hashimoto et al., the follow-up period ranged from seven months to 20 years [13].

This case underscores the subtle manifestation of intracranial IPEH characterized by localized scalp swelling and bony erosion, yet presenting without neurological symptoms, complicating radiological differentiation from other vascular lesions or bone pathologies. It is important to recognize that the radiological differential diagnosis encompasses vascular lesions such as angiosarcoma and Kaposi sarcoma, which might necessitate further interventions.

## Conclusions

Our patient was diagnosed with Masson's tumor, which caused destruction of the bone in the frontal region and resulted in epidural compression. Although the diagnosis was not predicted radiologically and macroscopically, it was confirmed through pathological examination. It is important to remember that in instances where the radiological diagnosis is uncertain, Masson's tumor should be included in the differential diagnosis for cases featuring cranial bone erosion, and that recurrence is unavoidable if the lesion is not fully excised. Total resection was necessary for the surgery, and no recurrence was detected during the early follow-up appointments. The patient underwent long-term monitoring to ensure the success of the procedure.
